# Antigen Positivity Without Infection: Confirmatory Mapping in Children Rules out Lymphatic Filariasis in Loiasis Co-Endemic Settings in Gabon

**DOI:** 10.3390/tropicalmed11100261

**Published:** 2026-09-23

**Authors:** Didier Bakajika, Julienne Atsame, Jeremie Rody Pambou, Philemon Koumba Koumba, Sylvie Alberte Ntsame Ella, Rychy Fustel Kombila Kombila, Davi Mawule, Ghislaine Nkone Asseko, Kelly Zongo, Mamadou Serme, Ambroise Bonkoungou, Sanon Karim, Moussa Sounkalo Sanfo, Aime Adjami, Dyesse Yumba, Honorat Zouré, Jorge Cano, Elizabeth Juma

**Affiliations:** 1 Expanded Special Project for Elimination of Neglected Tropical Diseases (ESPEN), World Health Organization Regional Office for Africa, Brazzaville P.O.Box 06, Congo; ambroise.bonkoungou@who.int (A.B.); sanonk@who.int (S.K.); sanfomo@who.int (M.S.S.); adjamia@who.int (A.A.); zoureh@who.int (H.Z.); canoj@who.int (J.C.); jumae@who.int (E.J.); 2 Programme for the Control of Parasitic Diseases, Ministry of Health, Libreville P.O.Box 820, Gabon; julienneatsame@yahoo.fr (J.A.); jeremyskol4@gmail.com (J.R.P.); kkoumbaphilemon@gmail.com (P.K.K.); sntsameella@gmail.com (S.A.N.E.); rychykombila4@gmail.com (R.F.K.K.); 3 World Health Organization Country Office, Libreville P.O.Box 820, Gabon; m.davime@gmail.com (D.M.); nkoneassekog@who.int (G.N.A.); 4 The END Fund, New York, NY 10016, USA; kelly.gillin@gmail.com; 5 National Neglected Tropical Diseases Control Programme, Ministry of Health, Ouagadougou 03 BP 7009, Burkina Faso; sermemamadou@gmail.com

**Keywords:** lymphatic filariasis, confirmatory mapping, Gabon, *Loa loa*, cross-reactivity, mass drug administration, QFAT, *Wuchereria bancrofti*

## Abstract

Precise mapping is essential for guiding lymphatic filariasis (LF) elimination programmes, yet antigen tests in loiasis co-endemic settings must be read with caution because of cross-reactivity. Gabon’s 2015 national mapping flagged 18 of 52 health districts (HDs) as potentially endemic, but that signal needed confirmation before committing populations to mass drug administration (MDA). We surveyed all 18 HDs between July and August 2025, enrolling 6688 children aged 9–14 years from 341 communities and testing them with the STANDARD™ Q Filariasis Antigen Test (QFAT); every QFAT-positive child then underwent nocturnal calibrated thick-smear microscopy and Wb-LDR quantitative PCR (qPCR) on dried blood spots. Thirteen children (0.19%) were QFAT-positive across eight HDs, and 17 of 18 HDs fell below the confirmatory mapping threshold for MDA. None of the thirteen carried *Wuchereria bancrofti* microfilariae or DNA; microscopy instead identified *Loa loa* in three and *Mansonella perstans* in one. MDA for LF is not warranted in the surveyed HDs, and these findings show why antigen results in loiasis co-endemic settings require parasitological or molecular confirmation before any programmatic decision.

## 1. Introduction

Lymphatic filariasis (LF) is a mosquito-borne neglected tropical disease caused mainly by *Wuchereria bancrofti* in sub-Saharan Africa. Chronic infection can lead to lymphoedema, elephantiasis and hydrocele, with substantial disability, stigma and socioeconomic loss for affected individuals and communities [1,2]. Globally, hundreds of millions of people remain at risk in countries where elimination of LF as a public health problem has not yet been validated, and the World Health Organization (WHO) road map for neglected tropical diseases targets elimination by 2030 [3,4,5].

Accurate mapping is the foundation of LF elimination programmes because it determines where mass drug administration (MDA) should be implemented [6]. WHO guidance recommends the use of circulating filarial antigen tests to identify areas where *W. bancrofti* infection is likely present and to support programmatic decisions on preventive chemotherapy [6,7]. In regions of uncertain endemicity, a confirmatory mapping framework was established to facilitate probability-based testing among children aged 9–14 years; this approach utilizes a critical-value decision rule to classify implementation units against a 2% antigen prevalence threshold while controlling for Type I and Type II error rates [8]. Within this framework, health districts (HDs) with fewer than three positive rapid diagnostic test results within the target sample are classified as falling below the threshold for MDA, whereas three or more positive results necessitate further programmatic interpretation [8].

In settings where suspected LF overlaps with loiasis, interpretation of circulating filarial antigen test results requires caution [9]. Filarial antigenaemia in the absence of confirmed *W. bancrofti* infection was first documented in the Democratic Republic of the Congo in an area without bancroftian filariasis but with *L. loa* night-blood microfilaraemia [10]. Subsequent studies confirmed that antigen tests used for LF diagnosis, in particular the BinaxNOW Filariasis immunochromatographic test (ICT; Abbott Diagnostics Scarborough, Inc., Scarborough, ME, USA) and the Filariasis Test Strip (FTS, Alere Inc., Scarborough, ME, USA; now Abbott), may yield positive results in individuals without *W. bancrofti* infection but with high *L. loa* microfilaraemia [11]. Mapping in Cameroon further demonstrated that areas initially suspected of LF endemicity may show no evidence of *W. bancrofti* when confirmatory strategies account for loiasis [12], and laboratory investigations have identified specific *L. loa* antigens responsible for cross-reactivity with rapid diagnostic tests for LF [13]. This body of evidence has been comprehensively summarized in a regional review of the apparent low prevalence of LF in high-risk *L. loa* areas of Central and West Africa [14].

Gabon is co-endemic for *L. loa* and *M. perstans*, with previously reported nationwide prevalences of 22.4% and 10.2%, respectively, in individuals over 15 years of age and significantly higher prevalence in forest ecosystems [15]. However, the interpretation of these findings remained ambiguous: in several communities, target sample sizes were not met, necessitating the grouping of communities during implementation. Additionally, the potential for cross-reactivity in antigen tests in areas where suspected LF overlaps with loiasis raised concerns that some positive results might not indicate true *W. bancrofti* infection.

To address these uncertainties, the Regional Programme Advisory Group for Preventive Chemotherapy Neglected Tropical Diseases (PC-NTD RPAG) in the WHO African Region recommended confirmatory mapping in the 18 HDs previously classified as potentially endemic. The present study had two objectives: (i) to determine whether each of these 18 HDs met the WHO confirmatory mapping decision rule for LF MDA based on STANDARD™ Q Filariasis Antigen Test (QFAT) testing in children aged 9–14 years and (ii) to determine whether QFAT-positive results were supported by parasitological evidence of microfilaraemia and by molecular evidence of *W. bancrofti* DNA among the antigen-positive children.

## 2. Materials and Methods

### 2.1. Study Design and Sampling Strategy

We conducted a cross-sectional descriptive epidemiological survey between July and August 2025. The primary objective was to determine whether the 18 HDs previously classified as potentially endemic for LF met confirmatory mapping criteria for MDA. A nested second objective was to determine whether QFAT-positive antigen results were supported by parasitological microscopy and molecular quantitative polymerase chain reaction (qPCR) evidence of *W. bancrofti* infection. Examination for chronic clinical manifestations compatible with LF (leg and scrotal swelling) was conducted in parallel at the household level among enrolled children and any adults present in their households.

The investigation encompassed 18 HDs across seven health regions in Gabon, all of which were identified as potentially endemic during the 2015 mapping initiative and consequently selected for confirmatory assessment by the national programme. In this study, the HD, or department, was the implementation unit, and the community was the settlement from which participants were drawn; a cluster refers to a community selected for the sample. The target cohort consisted of children aged 9–14 years who had resided in their respective communities for at least six months, a criterion established to ensure sufficient exposure to local epidemiological conditions while minimizing potential biases associated with population mobility.

Two sampling strategies were applied according to the number of communities within each HD, following the WHO confirmatory mapping protocol [6]. For departments comprising fewer than 30 communities, all communities were surveyed, and households were selected systematically to achieve a target sample size of at least 320 children per department. In departments consisting of 30 or more communities, 30 communities were selected as clusters using probability proportional to population size; within each cluster, households were selected randomly, and approximately 16 eligible children were enrolled, yielding a target sample size of at least 480 children per department. These sample size targets adhere to the critical-value framework established for confirmatory mapping against a 2% antigen prevalence threshold [8].

Two procedural deviations from the study design warrant acknowledgement. First, the assignment of sampling strategies was based on programmatic community lists and demographic estimates that were not consistently up to date. In Mulundu, although cluster sampling was intended, field implementation revealed a discrepancy between the projected and actual number of accessible, eligible communities. Consequently, systematic sampling was employed to complete the survey within the logistical and temporal constraints. Second, in two smaller departments—Louetsi–Bibaka and Mougalaba—the available cohort of eligible 9–14-year-olds was insufficient to meet the protocol-defined target of 320 children, yielding final sample sizes of 235 and 175, respectively. These deviations are further elaborated upon in the “Limitations” subsection of the Discussion.

### 2.2. Antigen Detection

Circulating filarial antigen was detected using the QFAT (SD Biosensor, Suwon, Republic of Korea), a lateral-flow rapid diagnostic test for *W. bancrofti* antigen [16,17,18]. Twenty microlitres of capillary blood obtained by finger-prick was applied to the sample port, and the test was run with chase buffer according to the manufacturer’s instructions. Results were read by trained field staff at 10 min under daylight. Any QFAT yielding an invalid result was repeated using a fresh device, and the result of the repeat test, whether positive or negative, was recorded as the final result for that participant. Test kits were stored between 2 and 30 °C in accordance with the manufacturer’s instructions. All field staff received centralized training from the national programme prior to deployment using standard photographic references for line interpretation.

### 2.3. Detection of Microfilaraemia

Every child with a positive QFAT underwent a single nocturnal capillary blood sampling between 22:00 and 01:00 h, corresponding to the expected peak of nocturnal *W. bancrofti* microfilaraemia [6,19]. Sixty microlitres of capillary blood were collected using a calibrated capillary tube and prepared as thick blood smears on microscope slides. Smears were air-dried, dehaemoglobinized in water, fixed with methanol and stained with Giemsa according to standard parasitological procedures. Slides were examined under light microscopy at ×100 and ×400 magnification by experienced laboratory technicians; positive or doubtful slides were re-read independently by a senior technician, with morphological identification of microfilariae confirmed under oil immersion.

### 2.4. DNA Extraction and qPCR

Dried blood spots (DBSs) from all QFAT-positive children were prepared for molecular confirmation. Approximately 100 µL of capillary blood was deposited onto filter paper, allowed to dry at ambient temperature, stored in sealed bags containing desiccants, and preserved at −20 °C until laboratory analysis. Genomic DNA was extracted using the DNeasy Blood & Tissue Kit (Qiagen, Hilden, Germany). One to two 6 mm punches were excised from each DBS and incubated in 180 µL of ATL buffer and 20 µL Proteinase K at 56 °C for 3–4 h or overnight if required. Following lysis, 200 µL AL buffer was added, and the samples were incubated at 70 °C for 10 min. DNA was precipitated with 200 µL ethanol and purified using DNeasy spin columns according to the manufacturer’s instructions. DNA was eluted in 50 µL AE buffer and stored at −20 °C until analysis.

Detection of *W. bancrofti* DNA was performed using a TaqMan real-time PCR assay targeting the *Wb*-LDR repeated sequence, with forward primer Wb-LDR-F (5′-CCAGCAGCCGCGGTAAT-3′), reverse primer Wb-LDR-R (5′-CGCCCTTTACGCCCAAT-3′) and probe Wb-LDR-P (5′-FAM-CGGAGAGGGCTAGCGTTATTCGGAATT-TAMRA-3′), as previously described by Rao and colleagues [20]. Amplification was carried out using Rotor-Gene Q Real-Time PCR System, 5-Plex HRM model (Qiagen, Hilden, Germany; Serial No. 1222109), with initial denaturation at 95 °C for 10 min, followed by 45 cycles of 95 °C for 15 s and 60 °C for 1 min. Each run included a positive control containing the *Wb*-LDR target sequence, a no-template negative control and an extraction-blank control to ensure assay validity. Data acquisition and analysis were performed using Rotor-Gene Q Software version 2.3.5 (Qiagen, Hilden, Germany).

### 2.5. Clinical Morbidity Assessment

Assessment for chronic manifestations compatible with LF was conducted at the household level by trained field teams accompanied by community health workers: in each household where a child was enrolled and gave blood, any adults present were also examined, together with the enrolled children. Swelling of the legs and, where present, of the upper limbs and genital area was recorded on visual inspection, with reference to the standard WHO clinical staging for lymphoedema. Scrotal swelling was recorded by visual inspection of the scrotum among males. These signs are clinically compatible with lymphoedema and hydrocele, respectively; they were documented descriptively and were not subjected to aetiological confirmation during the survey.

### 2.6. Data Collection and Management

Data were collected using paper-based forms and the ESPEN Collect mobile application (https://espen.afro.who.int/tools-resources/data-collection-tools/espen-collect, accessed on 15 June 2026). Each field team was supervised by a designated supervisor responsible for ensuring adherence to survey procedures. Central coordination was provided by the National Programme for the Control of Parasitic Diseases in collaboration with WHO and technical partners. Data quality was monitored daily by a programme data manager, who performed data cleaning and verification.

### 2.7. Statistical Analysis

Data were entered and managed in Microsoft Excel (Microsoft Corp., Redmond, WA, USA). Descriptive statistics summarized the number of children tested, QFAT-positive results, microscopy results, qPCR results and morbidity findings. Antigen prevalence and 95% confidence intervals (CI) were computed at the HD level using the Wilson score interval. Interpretation of confirmatory mapping results was conducted at the HD level: departments with fewer than three positive rapid diagnostic test results were interpreted as below the threshold requiring MDA, while three or more positive results required further programmatic review [21]. Microscopy and *Wb*-LDR qPCR results were then used to assess whether QFAT-positive antigen results were supported by parasitological or molecular evidence of *W. bancrofti* infection.

## 3. Results

### 3.1. Survey Implementation and Study Population

The confirmatory mapping survey was conducted between July and August 2025 in 18 HDs across seven health regions of Gabon. A total of 6688 children aged 9–14 years from 341 communities had an interpretable QFAT result and were included in the analysis. Sampling was implemented using systematic or cluster sampling according to the number of communities in each HD, with the protocol deviation in Mulundu noted in the Methods. One initial QFAT result was invalid and was repeated using a fresh device per protocol; the repeat result was used as the final result for that participant and is reflected in the analysis.

### 3.2. Antigen Detection and Confirmatory Mapping Interpretation

Thirteen children tested positive for circulating filarial antigen, giving an overall antigen prevalence of 0.19% (95% CI 0.11–0.33%). Among antigen-positive children, 10 were male and three were female. QFAT-positive children were identified in eight HDs. No antigen-positive children were detected in the remaining 10 HDs. Of the eight departments with at least one QFAT-positive child, seven recorded only one or two positives and therefore remained below the confirmatory mapping threshold; only Mulundu, with five positives, exceeded it. The 17 departments below the threshold thus comprise the 10 departments with no positives (0% prevalence), together with these seven departments with one or two positives.

Seventeen of the 18 surveyed HDs had fewer than three positive QFAT results and were therefore below the WHO confirmatory mapping threshold for MDA. Mulundu was the only HD exceeding the threshold, with five QFAT-positive children among 479 tested (1.04%; 95% CI 0.45–2.42%) and therefore required further programmatic interpretation (Table 1). The spatial distribution of QFAT-positive results across the 18 surveyed HDs is shown in Figure 1.

Health departments are shaded by QFAT result against the WHO confirmatory mapping decision rule: above threshold (≥3 QFAT-positive children; orange), below threshold (<3 QFAT-positive children; green) and not surveyed (grey). No QFAT-positive children in any department had parasitological or molecular evidence of *W. bancrofti* infection.

### 3.3. Parasitological and Molecular Validation

All 13 QFAT-positive children underwent nocturnal calibrated thick-smear microscopy and had DBS samples analysed by *Wb*-LDR qPCR. No *W. bancrofti* microfilariae were detected on any blood smear. Four slides were positive for non-LF filariae: three with microfilariae morphologically consistent with *L. loa* and one with *M. perstans*. The remaining nine slides were negative for microfilariae. Microfilarial densities on the four positive slides were low, ranging from 1 to 10 microfilariae per slide (*L. loa*: 10 in Louétsi–Bibaka, and three and one in Mulundu; *M. perstans*: five in Mulundu). These slides were independently re-read and counter-read at the ESPEN reference laboratory in Ouagadougou, which confirmed the absence of *W. bancrofti* and the low-density non-lymphatic filarial species (Supplementary Table S1). All 13 DBS samples were negative for *W. bancrofti* DNA by *Wb*-LDR qPCR. Thus, none of the QFAT-positive children had parasitological or molecular confirmation of *W. bancrofti* infection (Table 2).

In Mulundu specifically, where five QFAT-positive children were identified, none had *W. bancrofti* microfilariae on nocturnal microscopy, and all corresponding DBS samples were qPCR-negative. Instead, microscopy detected *L. loa* in two children and *M. perstans* in one child from this HD.

### 3.4. Clinical Morbidity Findings

Household examination identified 51 individuals with leg swelling and 54 with scrotal swelling across the 18 surveyed HDs (Table 3). Most occurred in adults: 38 of the 51 with leg swelling were adults (aged 29–81 years, median 60) and 13 were in the 9–14-year child cohort, while 38 of the 54 with scrotal swelling were adults (aged 16–90 years, median 55) and 16 were children. The distribution was heterogeneous. The highest number of scrotal-swelling cases was reported in Ogoulou (n = 14), followed by Lopé (n = 10). The highest numbers of leg-swelling cases were observed in Ivindo (n = 10) and Abanga–Bigné (n = 9); Abanga–Bigné also reported four scrotal-swelling cases. Although leg and scrotal swelling are compatible with LF morbidity (lymphoedema and hydrocele), the aetiology of these manifestations was not confirmed in this survey. The predominance of these chronic manifestations in adults is consistent with cumulative lifetime exposure; where leg swelling reflects chronic lymphatic disease in particular, the causative parasite has frequently cleared by the time disease is established, so a negative parasitological result in a child sentinel survey can neither establish nor exclude past *W. bancrofti* infection in these adults. Aetiological attribution would require individual clinical–epidemiological assessment—length of residence in an endemic area, disease progression and exclusion of non-filarial causes—which was beyond the scope of this mapping survey.

## 4. Discussion

In this confirmatory mapping survey, QFAT antigen positivity was not corroborated by either nocturnal microscopy or Wb-LDR qPCR, indicating that the antigen signal did not reflect active *W. bancrofti* infection. Among the 6688 children tested, only 13 (0.19%) were antigen-positive; 17 of the 18 HDs fell below the confirmatory mapping threshold, and Mulundu was the sole department above it. Critically, none of the 13 antigen-positive children, including the five from Mulundu, harboured *W. bancrofti* microfilariae or DNA. In contrast, microscopy identified *L. loa* in three children and *M. perstans* in one. Taken together, these results indicate that the QFAT-positive findings were not supported by parasitological or molecular evidence of *W. bancrofti* and that MDA for LF is not warranted in the surveyed departments of Gabon.

Our observations align with the established body of evidence concerning antigen test cross-reactivity in settings co-endemic for loiasis. The phenomenon of filarial antigenaemia occurring without confirmed *W. bancrofti* infection was initially reported in the Democratic Republic of the Congo, in a region where bancroftian filariasis is absent but nocturnal *L. loa* microfilaraemia is present [9,10,22]. Pion and colleagues subsequently showed that antigen tests used for LF diagnosis may be positive in individuals without *W. bancrofti* infection but with high *L. loa* microfilaraemia [11]. Mapping in Cameroon, applying a confirmatory algorithm that accounted for loiasis, found no evidence of *W. bancrofti* endemicity [12]. However, Hertz and colleagues identified specific *L. loa* antigens responsible for cross-reactivity with rapid diagnostic tests for LF [13]. A regional review by Kelly-Hope and colleagues consolidated evidence of low LF prevalence in high-risk *L. loa* areas of Central and West Africa [14]. The present study extends this evidence to Gabon and to the newer QFAT platform and supports the conclusion that antigen positivity in loiasis co-endemic settings should not be interpreted as confirmation of LF infection without parasitological or molecular support.

The detection of *L. loa* in three children and *M. perstans* in one child from among the 13 QFAT-positive children is biologically plausible in the Gabonese context. Rural areas were documented to have 20.4% prevalence for *L. loa* and 10.2% for *M. perstans*, with significantly higher prevalence in forest ecosystems [23]. Children aged 9–14 years are expected to have lower prevalences than adults, but exposure is already substantial in this age band in forest communities, which fits the observed pattern [24]. The absence of *W. bancrofti* by both microscopy and qPCR, combined with the detection of non-LF filariae in nearly one-third of the antigen-positive children, supports the interpretation that the QFAT-positive results in this survey reflect either cross-reactive antigenaemia with co-endemic filariae or other non-*W. bancrofti* antigen reactivity, rather than active LF infection.

Consistent with this, all eight departments in which QFAT positivity was recorded lie within loiasis-hyperendemic implementation units: the rapid assessment procedure for loiasis (RAPLOA) predicts *L. loa* prevalence of approximately 51–83% across these departments (WHO/ESPEN; Supplementary Figure S1), well above the 40% threshold that defines high-risk loiasis communities [25,26]. The concentration of antigen positivity within the most intensely loiasis-endemic departments provides an ecological basis for interpreting the QFAT signal as cross-reactive rather than indicative of *W. bancrofti* transmission. We note, however, that the observed microfilarial densities were low, so mechanisms beyond density-dependent cross-reactivity—including cross-reactive *L. loa* antigens and, as flagged by the reference laboratory, occasional slide artefacts mistaken for microfilariae—may also contribute.

The combined use of QFAT, nocturnal microscopy and *Wb*-LDR qPCR strengthens the interpretation of the confirmatory mapping results. While antigen testing provides a practical field-based screening tool, microscopy and molecular testing add diagnostic specificity in settings where other filarial infections may interfere with interpretation. The *Wb*-LDR qPCR assay targets a repeated DNA sequence and is a sensitive molecular method for detecting *W. bancrofti* DNA in blood and mosquitoes [20,27,28]. The negative qPCR results across all 13 antigen-positive children, therefore, provide important confirmatory evidence against active *W. bancrofti* infection.

We acknowledge that circulating filarial antigen can occur without detectable microfilariae in genuine *W. bancrofti* infection—during the prepatent period, with single-sex or all-male worm burdens, or in amicrofilaraemic “endemic normal” individuals—so antigen results should not be over-interpreted in either direction. Our interpretation does not, however, rest on antigen testing alone: the concurrent negativity of *Wb*-LDR qPCR, which detects parasite DNA independently of microfilarial periodicity, together with the identification of alternative filarial aetiologies and independent reference-laboratory confirmation, makes active bancroftian infection an unlikely explanation for the antigen signal. We nonetheless recognize that blood-based qPCR may not capture adult worms sequestered in the lymphatics that shed little DNA into peripheral blood, and that antigen negativity alone is likewise insufficient to justify stopping mass drug administration; both directions require the confirmatory framework applied here rather than any single test.

Several limitations should be acknowledged. First, the survey did not include entomological xenomonitoring [6]. The findings should therefore be interpreted as evidence for LF endemicity classification at the human-host level rather than as a direct measurement of vector-borne transmission. Second, microscopy and qPCR were performed only among QFAT-positive children. This approach is consistent with reassessment strategies in which night blood smears are commonly performed among antigen-positive individuals because antigen-negative individuals have a low probability of detectable microfilaraemia. Nevertheless, this design cannot exclude occult, very-low-density microfilaraemia below the detection limits of microscopy and dried-blood-spot qPCR, whether among antigen-negative children or among the antigen-positive children themselves, in whom a truly low-level *W. bancrofti* infection cannot be formally excluded based on these assays alone. This residual possibility is, however, unlikely to alter the programmatic classification: all 13 antigen-positive children were negative by two independent methods and by an independent reference-laboratory read, and department-level positive counts remained below the decision threshold in every department except Mulundu, where all five positives were parasitologically and molecularly negative. Third, DBS qPCR for *W. bancrofti* has a sensitivity floor that may limit detection at very low microfilarial densities [29], although this is a fair limitation that strengthens rather than weakens a negative finding. Fourth, the survey sampled only children aged 9–14 years; if adult populations harbour a residual reservoir of infection in any of these departments, this would not be detected by the present design [30]. Fifth, two sample-size considerations affect interpretation: in two small HDs—Louetsi–Bibaka and Mougalaba—the eligible 9–14-year-old population available for enrolment was below the protocol target [6], and the achieved sample sizes were 235 and 175 children, respectively. Both departments had zero QFAT-positive results and would not be expected to cross the WHO threshold of three positives even at full target enrolment. Still, the statistical confidence around these negative classifications is correspondingly reduced. Sixth, the sampling strategy relied on programme community lists and population estimates that were not consistently up to date. In Mulundu, discrepancies between projected and accessible population counts led to a mid-implementation switch from cluster to systematic sampling. This limitation should be considered when interpreting the Mulundu-specific estimates and underscores the necessity of using up-to-date community-level data for future surveys. Finally, morbidity data were descriptive and not linked to aetiological confirmation; therefore, leg and scrotal swelling should not be interpreted as evidence of active *W. bancrofti* infection. In the absence of supportive parasitological or molecular evidence among QFAT-positive children, these findings should be interpreted cautiously and should not be used alone to infer active LF endemicity. These morbidity findings were, moreover, ascertained opportunistically among individuals present in the households of enrolled children rather than through a systematic community census and therefore do not constitute population-based prevalence.

From a programmatic standpoint, two recommendations follow. First, based on the combined antigen, microscopy and molecular evidence—particularly given the known cross-reactivity of antigen tests in loiasis co-endemic settings [11,13,14]—LF MDA is not warranted in the 18 surveyed HDs of Gabon. Although Mulundu exceeded the QFAT-based decision threshold, the definitive absence of confirmatory *W. bancrofti* evidence by both microscopy and highly sensitive qPCR [27,28] strongly argues against initiating MDA based on antigen positivity alone. We recommend that Mulundu be retained under enhanced surveillance, with consideration of a repeat QFAT survey, expanded sampling that includes adults, and entomological xenomonitoring if vector populations are present. Second, our experience strongly supports the integration of confirmatory testing into LF mapping protocols in loiasis co-endemic settings while acknowledging that the logistical demands of nocturnal blood collection render nocturnal blood microscopy infeasible for some programmes. When feasible, confirmatory testing should ideally include both parasitological microscopy on nocturnal blood and molecular testing on DBS. In settings where nocturnal blood collection is not operationally viable, programmes should prioritize alternative complementary lines of evidence, such as molecular confirmation on DBS collected during routine antigen testing, targeted referral of antigen-positive individuals for secondary evaluation, or entomological xenomonitoring. The selection of a specific confirmatory algorithm must be tailored to local resources and capacity. For LF mapping in loiasis co-endemic settings of Central Africa, we suggest a practical sequence to minimize diagnostic misclassification: (i) map loiasis endemicity (for example by RAPLOA) alongside LF so that departments at high risk of antigen cross-reactivity are identified in advance; (ii) collect dried blood spots for molecular confirmation at the time of antigen testing, which avoids the logistical burden of nocturnal blood collection; (iii) subject a sample of slides to independent reference-laboratory reading and counter-reading for quality assurance, given the false positives that poor slide preparation can generate; and (iv) reserve nocturnal microscopy and entomological xenomonitoring for departments that exceed the antigen threshold. The cost-effectiveness of this strategic approach has been established in Ethiopia and the United Republic of Tanzania, where the avoidance of unnecessary MDA across 52 of 55 districts yielded substantial programmatic savings [8,21]. Our findings demonstrate that this evidence base extends to the Gabonese context.

Regarding clinical manifestations, the documented cases of leg and scrotal swelling (clinically compatible with lymphoedema and hydrocele) underscore an urgent, continuing need to strengthen morbidity management and disability prevention (MMDP) services regardless of the LF endemicity classification. These manifestations represent a significant existing health burden; therefore, affected individuals must continue to receive appropriate clinical assessment, comprehensive lymphoedema care and access to hydrocele surgery where indicated, irrespective of ongoing programmatic elimination activities.

## 5. Conclusions

This confirmatory mapping survey found no parasitological or molecular evidence of *W. bancrofti* infection across 18 HDs of Gabon despite some areas initially exceeding antigen-based decision thresholds. The comprehensive analysis, including microscopy and highly sensitive qPCR, strongly indicates that LF MDA is not warranted in the surveyed areas, particularly given the known cross-reactivity of antigen tests in *L. loa* co-endemic settings. Therefore, confirmatory parasitological and/or molecular testing must accompany antigen-based mapping in *L. loa* co-endemic countries to avoid misclassification and prevent unnecessary MDA, a strategy that has proven cost-effective and has led to substantial programmatic savings in other contexts. Furthermore, MMDP services must continue regardless of endemicity status, as existing cases of leg and scrotal swelling represent a significant and continuing health burden requiring appropriate clinical care.

This classification is made at the level of the child sentinel population within the WHO confirmatory mapping framework; because the survey did not include adults or entomological xenomonitoring, it should be read as evidence against the need for MDA rather than as definitive proof of interrupted transmission. Mulundu in particular warrants enhanced surveillance—including adult sampling and xenomonitoring—before a final endemicity classification.

## Figures and Tables

**Figure 1:**
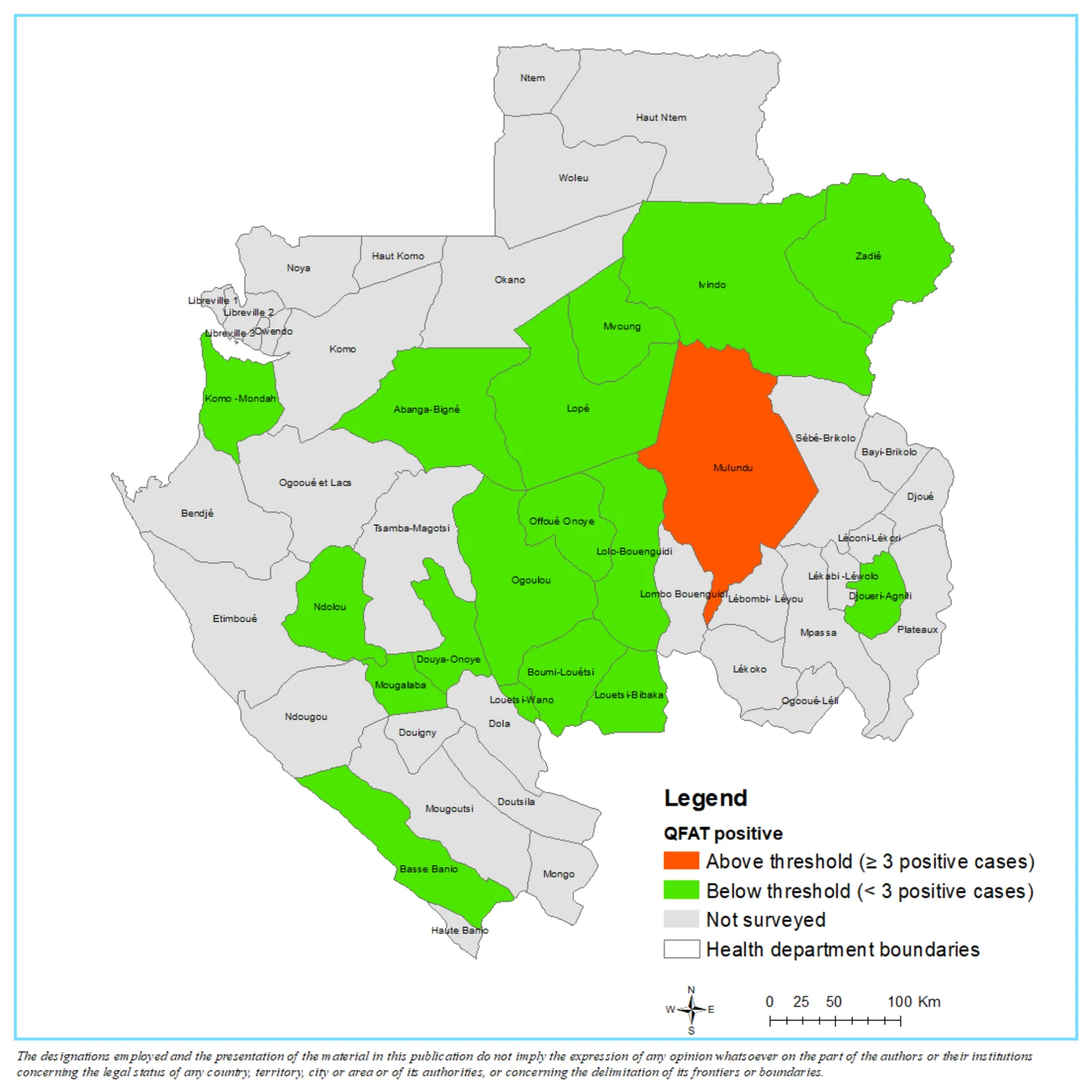
Confirmatory mapping of lymphatic filariasis in 18 health departments of Gabon, July–August 2025.

**Table 1:** Distribution of QFAT-positive children by health department, with confirmatory mapping interpretation against the WHO decision rule.

Region	Health Department	Children Tested (n)	QFAT-Positive (n)	Prevalence % (95% CI)	Interpretation
Centre	Abanga–Bigné	500	1	0.20 (0.04–1.12)	Below threshold
Centre–Est	Lolo–Bouenguidi	484	1	0.21 (0.04–1.16)	Below threshold
Centre–Est	Mulundu	479	5	1.04 (0.45–2.42)	Above threshold
Centre–Est	Offoué–Onoye	304	1	0.33 (0.06–1.84)	Below threshold
Centre–Sud	Boumi-Louetsi	346	0	0.00 (0.00–1.10)	Below threshold
Centre–Sud	Douya–Onoye	446	0	0.00 (0.00–0.85)	Below threshold
Centre–Sud	Louetsi–Bibaka	235	1	0.43 (0.08–2.37)	Below threshold
Centre–Sud	Louetsi–Wano	344	0	0.00 (0.00–1.10)	Below threshold
Centre–Sud	Mougalaba	175	0	0.00 (0.00–2.15)	Below threshold
Centre–Sud	Ndolou	342	0	0.00 (0.00–1.11)	Below threshold
Centre–Sud	Ogoulou	350	0	0.00 (0.00–1.09)	Below threshold
Est	Ivindo	512	0	0.00 (0.00–0.74)	Below threshold
Est	Lopé	349	1	0.29 (0.05–1.60)	Below threshold
Est	Mvoung	328	0	0.00 (0.00–1.16)	Below threshold
Est	Zadié	346	1	0.29 (0.05–1.62)	Below threshold
Ouest	Komo–Mondah	501	2	0.40 (0.11–1.44)	Below threshold
Sud	Basse–Banio	327	0	0.00 (0.00–1.16)	Below threshold
Sud–Est	Djouori–Agnili	320	0	0.00 (0.00–1.19)	Below threshold
Total	18	6688	13	0.19 (0.11–0.33)	**—**

A department was classed below the threshold when the number of QFAT-positive children fell below the critical value defined for its sample size, the level below which mass drug administration is not indicated [8]. Prevalence and 95% confidence intervals were computed using the Wilson score interval and are shown for transparency only; they were not used for classification. QFAT, STANDARD™ Q Filariasis Antigen Test; WHO, World Health Organization; MDA, mass drug administration.

**Table 2:** Parasitological microscopy and Wb-LDR qPCR results among QFAT-positive children by health district.

Health District	QFAT-Positive (n)	Smears Positive for Microfilariae (n)	Species Identified by Microscopy	Wb-LDR qPCR-Positive (n)
Komo–Mondah	2	0	None	0
Abanga–Bigné	1	0	None	0
Lopé	1	0	None	0
Zadié	1	0	None	0
Lolo–Bouenguidi	1	0	None	0
Mulundu	5	3	*L. loa* (n = 2); *M. perstans* (n = 1)	0
Offoué–Onoye	1	0	None	0
Louetsi–Bibaka	1	1	*L. loa* (n = 1)	0
Total	13	4	*L. loa* (n = 3); *M. perstans* (n = 1)	0

Microscopy was performed on nocturnal calibrated thick blood smears examined by light microscopy, with positive or doubtful slides re-read by a senior technician. None of the 13 QFAT-positive children had *Wuchereria bancrofti* microfilariae on microscopy or *W. bancrofti* DNA by Wb-LDR qPCR. QFAT, STANDARD™ Q Filariasis Antigen Test; qPCR, quantitative polymerase chain reaction; Wb-LDR, *Wuchereria bancrofti* long DNA repeat.

**Table 3:** Clinical findings (leg swelling and scrotal swelling) documented during household examination, by health district.

Region	Health Department	Leg Swelling (n)	Scrotal Swelling (n)	Total (n)
Centre	Abanga–Bigné	9	4	13
Centre–Est	Lolo–Bouenguidi	1	6	7
Centre–Est	Mulundu	4	0	4
Centre–Est	Offoué–Onoye	0	0	0
Centre–Sud	Boumi-Louetsi	5	7	12
Centre–Sud	Douya–Onoye	1	7	8
Centre–Sud	Louetsi–Bibaka	0	0	0
Centre–Sud	Louetsi–Wano	0	0	0
Centre–Sud	Mougalaba	0	0	0
Centre–Sud	Ndolou	0	0	0
Centre–Sud	Ogoulou	0	14	14
Est	Ivindo	10	1	11
Est	Lopé	7	10	17
Est	Mvoung	3	1	4
Est	Zadié	1	1	2
Ouest	Komo–Mondah	8	2	10
Sud	Basse–Banio	2	1	3
Sud–Est	Djouori–Agnili	0	0	0
Total (18 HDs)		51	54	105

Leg-swelling and scrotal-swelling cases (clinically compatible with lymphoedema and hydrocele) were documented descriptively on visual inspection and were not subjected to aetiological confirmation; they should not be read as evidence of active *Wuchereria bancrofti* infection. HD, health district. Cases were recorded across all ages during household examination and were predominantly in adults (leg swelling: 38 adults, 13 children aged 9–14; scrotal swelling: 38 adults, 16 children).

## Data Availability

The datasets analysed during this study are the property of the Ministry of Health of Gabon and cannot be shared without its authorisation. Requests for access may be directed to the corresponding author, who will forward them to the Ministry of Health of Gabon for consideration; any data released will be de-identified, with no personal identifiers or participant names shared.

## Supplementary Materials

The following supporting information can be downloaded at https://www.mdpi.com/article/10.3390/tropicalmed11100261/s1. Figure S1: Number of QFAT-positive children by department in relation to RAPLOA-predicted *Loa loa* prevalence in the eight departments with QFAT-positive children; Table S1: Independent reference-laboratory validation, including read and counter-read, of the 13 QFAT-positive blood slides from the Gabon confirmatory mapping survey, ESPEN reference laboratory, Ouagadougou. April 2026.
