# Supplementary material for: Antigen Positivity Without Infection: Confirmatory Mapping in Children Rules out Lymphatic Filariasis in Loiasis Co-Endemic Settings in Gabon
Source: Trop Med Infect Dis. 2026 Sep 23;11(10):261. doi: 10.3390/tropicalmed11100261 (PMC13709386; doi:10.3390/tropicalmed11100261)
Supplement: Supplementary file 1 [file tropicalmed-11-00261-s001.zip › tropicalmed-4448979-supplementary.pdf]

Supplementary Figure S1

Figure S1. Number of QFAT-positive children by department in relation to RAPLOA-predicted *Loa loa* prevalence in the eight departments with QFAT-positive children.

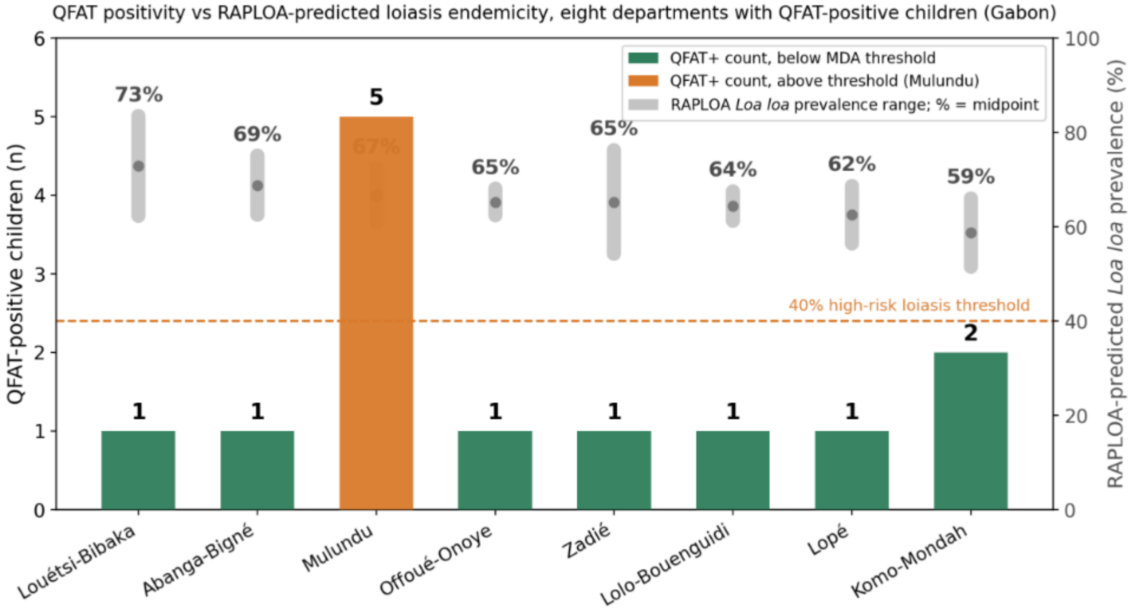

Bars show the number of QFAT-positive children per department (green, below the confirmatory mapping MDA threshold; orange, Mulundu, above threshold); grey bands show the department-level range of RAPLOA-predicted *Loa loa* prevalence (WHO/ESPEN), with the dashed line marking the 40% threshold that defines high-risk loiasis communities. All eight departments are loiasis hyper-endemic (approximately 51–83%). QFAT, STANDARD™ Q Filariasis Antigen Test; RAPLOA, rapid assessment procedure for loiasis; MDA, mass drug administration.

Supplementary Table S1

Table S1. Independent reference-laboratory validation, including read and counter-read, of the 13 QFAT-positive blood slides from the Gabon confirmatory mapping survey, ESPEN reference laboratory, Ouagadougou. April 2026

Slide	Department	Sex	Reference microscopy (Mf) result	Species identified	Microfilariae counted (mf)
S1	Komo-Mondah	M	Negative	—	—
S2	Komo-Mondah	M	Negative	—	—
S3	Lolo-Bouenguidi	F	Negative	—	—
S4	Louétsi-Bibaka	M	**Positive**	*Loa loa*	10
S5	Lopé	M	Negative	—	—
S6	Zadié	M	Negative	—	—
S7	Mulundu	M	Negative	—	—
S8	Mulundu	M	**Positive**	*Loa loa*	1
S9	Mulundu	M	**Positive**	*Loa loa*	3
S10	Mulundu	M	**Positive**	*Mansonella perstans*	5
S11	Mulundu	F	Negative	—	—
S12	Offoué-Onoye	F	Negative	—	—
S13	Abanga-Bigné	M	Negative	—	—

Slides were read and counter-read by two senior technicians following standardized procedures (10× survey of all three thick-smear bands; species confirmation at 100× under oil immersion). No *Wuchereria bancrofti* microfilariae were detected on any slide. Four slides were positive for non-lymphatic filariae only — *Loa loa* (n = 3) and *Mansonella perstans* (n = 1) — at low density (range 1–10 mf per slide). Participant identifiers have been removed; sex is recorded as reported on the slide label. Mf, microfilariae; QFAT, STANDARD™ Q Filariasis Antigen Test.
